# Gut microbiota of the critically endangered Saiga antelope across two wild populations in a year without mass mortality

**DOI:** 10.1038/s41598-023-44393-z

**Published:** 2023-10-11

**Authors:** Eveliina Hanski, Munib Khanyari, Jingdi Li, Kieran A. Bates, Steffen Zuther, Martin C. J. Maiden, Richard Kock, Sarah C. L. Knowles

**Affiliations:** 1 Department of Biology, University of Oxford, Oxford, UK; 2 Faculty of Medicine, University of Helsinki, Helsinki, Finland; 3 Nature Conservation Foundation, Mysore, India; 4 Association for the Conservation of Biodiversity of Kazakhstan, Astana, Kazakhstan; 5 Frankfurt Zoological Society, Frankfurt, Germany; 6 Centre for Emerging, Endemic and Exotic Diseases, The Royal Veterinary College, University of London, London, UK

**Keywords:** Conservation biology, Microbial ecology, Microbiology, Zoology

## Abstract

The Saiga are migratory antelopes inhabiting the grasslands of Eurasia. Over the last century, Saiga have been pushed to the brink of extinction by mass mortality events and intense poaching. Yet, despite the high profile of the Saiga as an animal of conservation concern, little is known of its biology. In particular, the gut microbiota of Saiga has not been studied, despite its potential importance in health. Here, we characterise the gut microbiota of Saiga from two geographically distinct populations in Kazakhstan and compare it with that of other antelope species. We identified a consistent gut microbial diversity and composition among individuals and across two Saiga populations during a year without die-offs, with over 85% of bacterial genera being common to both populations despite vast geographic separation. We further show that the Saiga gut microbiota resembled that of five other antelopes. The putative causative agent of Saiga mass die-offs, *Pasteurella multocida*, was not detected in the Saiga microbiota. Our findings provide the first description of the Saiga gut microbiota, generating a baseline for future work investigating the microbiota’s role in health and mass die-offs, and supporting the conservation of this critically endangered species.

## Introduction

**Figure 1:**
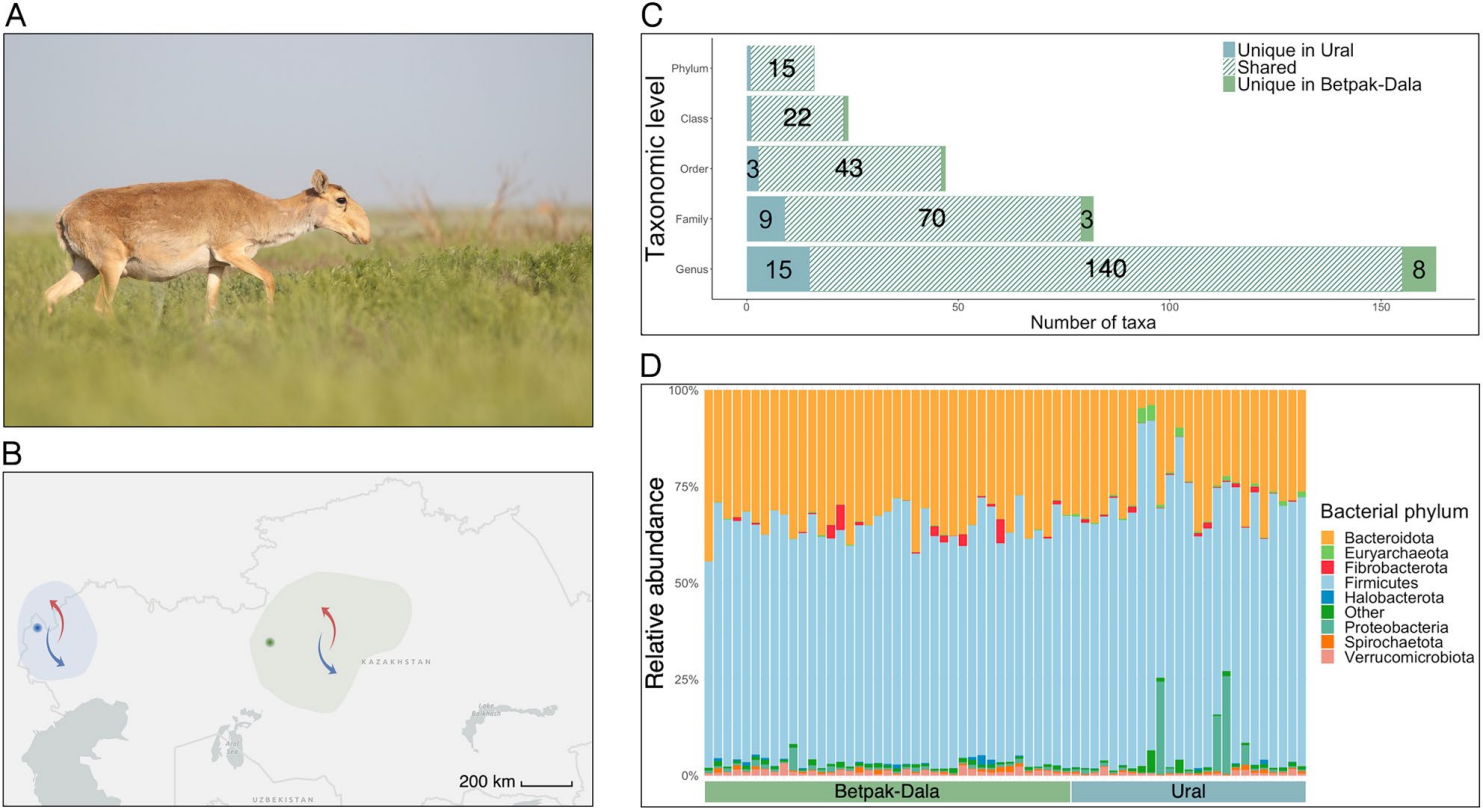
(**A**) A female Saiga antelope (*Saiga tatarica*). Photo credit: Albert Salemgareyev. (**B**) Geographic ranges of the two sampled Saiga antelope populations (green, Betpak-Dala population; blue, Ural population). Overall migration directions are indicated with arrows (red, migration direction in spring; blue, migration direction in autumn). Approximate centroids of faecal sample collection sites are indicated with dark dots within the habitats. (**C**) Number of shared and unique taxa between the two populations at five taxonomic levels. Numbers on bars indicate how many taxa were unique to Ural (blue), Betpak-Dala population (green), or that were shared between the two populations (striped teal). Number of unique/shared taxa is written when ≥ 3. (**D**) Gut microbiota composition of the Saiga antelope at phylum level. Rare taxa (mean relative abundance < 0.03% and prevalence < 0.10% across samples) are under ‘Other’. Stacked bars are individual samples. Horizontal bars indicate Saiga population.

The Saiga antelope (*Saiga tatarica* ssp.) is a long-distance migratory ungulate, famous for its distinctive pendulant nose (Fig. 1A). Once ranging across nearly the whole of Eurasia, the majority of Saiga now inhabit Kazakhstan, the largest landlocked country in the world. The Kazakh Saiga (*Saiga tatarica tatarica*) is of particular interest due to its vulnerability to mass mortality events, seemingly caused by *Pasteurella multocida*^1^. These mass mortality events, together with anthropogenic impacts such as poaching, have reduced the population of Kazakh Saiga by 95% in recent decades, from an estimated 1,200,000 to circa 50,000 between mid-1970s and 2003^2,3^. Since 2002, the Saiga has been listed as ‘critically endangered’ by the International Union for Conservation of Nature^3^; however, many aspects about the biology of the Saiga remain unknown. They live out of sight in the remote grasslands and deserts of Kazakhstan and have proved challenging to keep in captivity^4^, making this species particularly difficult to study. Since around the middle of the twentieth century, the Kazakh Saiga have been living as three geographically distinct populations in the Betpak-Dala, Ustyurt and Ural regions. Both the Betpak-Dala and Ural Saiga populations have suffered from mass die-offs, while residing a minimum of 500 km apart from each other (Fig. 1B).

A better understanding of the biology of the Saiga antelope could provide insights into their susceptibility to mass mortality events with potential conservation application. One key factor that can have important effects on mammal biology is the gut microbiota, the diverse community of microorganisms residing in the intestinal tract. While this community contains microorganisms that range from beneficial to pathogenic, as a whole it provides important functions for the host by regulating key physiological processes such as immune maturation and nutrient extraction^5,6^. Correspondingly, disruption of the gut microbiota can have adverse effects on the host and increase susceptibility to both infectious and non-infectious diseases^7^.

Many natural factors can influence gut microbiota composition in mammals, including diet, social interactions, infection, and aging^8–11^. A growing literature indicates how the microbiota of wild animals can be altered by changes to their environment such as exposure to chemicals, habitat destruction, infectious disease, urbanization, and housing in captivity^12–20^, yet knowledge about the implications for animal health remains limited. Given the lability and health impacts of the gut microbiota, an improved understanding of host–microbiota interactions also holds conservation potential^21,22^. For example, characterisation of the Saiga gut microbiota during a year without mass mortality will allow future investigation of the potential role of the microbiota in disease, and might inform the design of microbiota manipulation in captivity, e.g., through diet or faecal microbiota transplants, to facilitate captive breeding programmes for species recovery^21^.

Here, we provide the first characterisation of the Saiga gut microbiota during a year without die-offs, explore the extent of microbiota variation across two geographically distinct populations, and examine how the Saiga microbiota compares with that of other antelope species. These data provide a baseline understanding of the gut microbiota in this critically endangered species, which future work examining the potential significance of the microbiota for mass mortality events can build upon.

## Results

### Sequencing outcome of the Saiga antelope samples

A total of 80 faecal DNA samples from 70 Saiga antelope (Kazakhstan) (Fig. 1A, B) were sequenced using the Illumina MiSeq platform. Eight Saiga samples were lost during read depth filtering. The remaining 72 samples included eight duplicate samples (duplicate samples from a single faecal deposition, see “Methods”) that were sequenced to assess the robustness of our pipeline. Duplicates generally clustered together with their corresponding samples (Suppl. Fig. 1) and sample ID strongly predicted microbiota composition across these repeat samples, explaining 67.1% of all gut microbial variation (PERMANOVA based on Aitchison distance, *p* < 0.001), indicating the sampling method produced a repeatable representation of the faecal microbiota. These duplicates were removed from the dataset before further analysis. After filtering out singleton and doubleton amplicon sequence variants (ASVs) in the dataset, the two populations of Saiga collectively harboured a total of 4,036 unique ASVs, with a mean of 743 ASVs per sample (range 470–1057). Using the SILVA database (v.138.1), 92.9% ASVs could be assigned to family level, 74.7% to genus level, and 0.7% to species level. Due to the low level of assignment to species level, the highest taxonomic level we considered in analyses was genus.

### Composition and diversity of the Saiga antelope gut microbiota

Of the 4,036 ASVs found in Saiga, 2,689 (66.6%) were detected in both populations, while 570 (14.1%) were unique to the Ural population (*n* = 25) and 777 (19.3%) were unique to the Betpak-Dala population (*n* = 39). Across both populations, 16 bacterial phyla, 24 classes, 47 orders, 82 families, and 163 genera were identified (Fig. 1C). The two populations shared 15 out of 16 phyla (93.8%) and 91.5–91.7% of classes and orders. When inspecting gut microbiota profiles at the family and genus level, 85.4% and 85.9% of taxa were shared between the two populations, respectively. The shared bacterial families and genera formed 93.4–96.1% and 80.3–84.4%, respectively, of the total relative abundance in the Betpak-Dala and Ural populations, such that most bacteria in the Saiga microbiota belong to genera that are common to both populations. Unique genera in each population were not of any particular lower resolution taxa (such as family or class); instead, the Betpak-Dala population had 8 unique genera from 5 different classes. Similarly, the Ural population had 15 unique genera from 8 different classes.

At the phylum level, the Saiga gut microbiota was heavily dominated by Firmicutes and Bacteroidota, which together comprised 94.9% of all reads on average per sample (Fig. 1D). The Ural population harboured one unique phylum, Campilobacterota, that was rare and formed just 0.00002% of total abundance in this population. The shared microbiota (comprised of ASVs found in both populations, irrespective of relative abundance and prevalence) was proportionately dominated by the bacterial families Oscillospiraceae (20.8% and 16.6% of total relative abundance of taxa in Betpak-Dala and Ural, respectively) and Rikenellaceae (15.6% and 9.9%%), followed by 68 additional shared families (Suppl. Fig. 2). The shared microbiota contained 140 genera, with the predominant genus being Oscillospiraceae UCG-005 (16.9% and 11.8%). A small number of individuals across the two populations had an increased relative abundance of Proteobacteria (Fig. 1D), primarily driven by *Escherichia*/*Shigella* as revealed in the genus-level inspection of the Saiga gut microbiota (Suppl. Fig. 2).

Gammaproteobacteria, the class containing *P. multocida*, the bacterium implicated in Saiga mass mortalities^1^, was detected in both populations and formed 0.3% (Betpak-Dala) and 2.9% (Ural) of total abundance at class level; however, no ASVs were assigned to the family Pasteurellaceae nor the genus *Pasteurella.* Furthermore, a Nucleotide BLAST search was conducted for ASVs for which SILVA had either assigned the class Gammaproteobacteria or had failed to assign a class. This search did not produce a ≥ 98% species identity match for *P. multocida*, further confirming this bacterium was not detected in this Saiga gut microbiota dataset collected in a year without mass mortalities.

**Figure 2:**
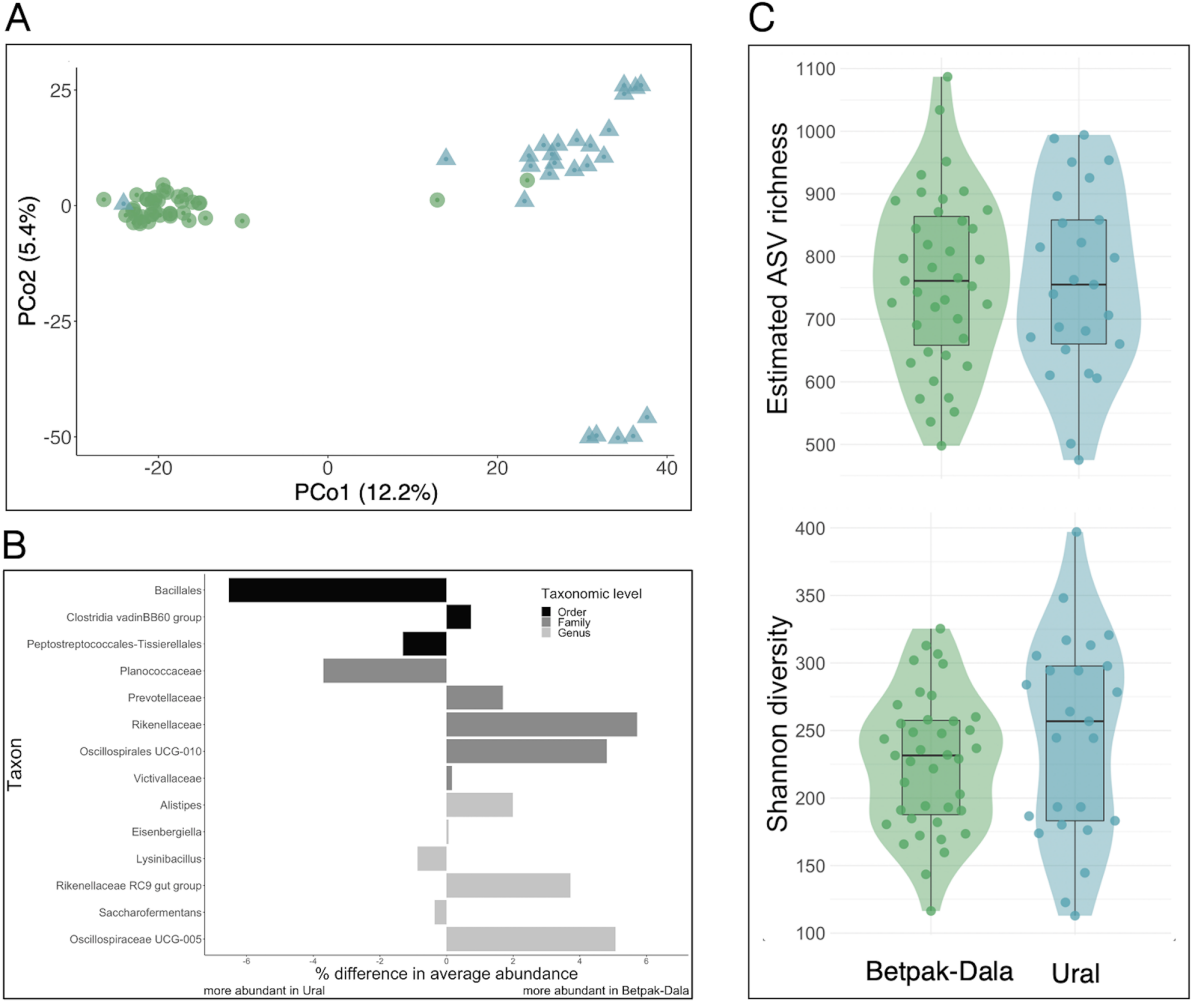
(**A**) Principal coordinate analysis of Betpak-Dala (green circles) and Ural (blue triangles) Saiga gut microbiota (dis)similarity based on Aitchison distance. Circles and triangles are individual samples. Microbiota similarity increases with proximity between sample points. (**B**) Bacterial taxa with significantly differing relative abundance between the Betpak-Dala and the Ural Saiga populations. Bars indicate the mean relative abundance in the Betpak-Dala population minus the mean relative abundance in the Ural population. Only taxa with a Benjamini–Hochberg corrected p-value of less than 0.05 and an effect size (standardized mean difference) greater than 1 are shown. (**C**) Sample-level asymptotic ASV richness (top) and Shannon diversity (bottom) of Betpak-Dala (green) and Ural (blue) Saiga. Circles are individual samples. Horizontal bar indicates median alpha diversity. Statistical differences between Betpak-Dala and Ural Saiga were tested with Wilcoxon rank sum tests (*p* > 0.05 for both ASV richness and Shannon diversity).

Despite the high proportion of shared ASVs across the two populations (shared ASVs formed 77.6% and 82.5% of all ASVs detected in the Betpak-Dala and Ural populations, respectively), the gut microbiota compositions of the two populations were detectably different, with 10.4% of variation in Aitchison dissimilarity being explained by population identity (PERMANOVA, p < 0.001; population identity explained 8.6% of Jaccard dissimilarity, *p* < 0.001). Samples clustered by population when ordinated with the exception of 3 samples, and these patterns were consistent across categorical (Aitchison, Jaccard) and phylogenetic distances (weighted and unweighted UniFrac) (Fig. 2A, Suppl. Fig. 3), indicating consistently that the two populations have distinct gut microbial communities. The three samples that did not cluster by populations were not the samples with increased relative abundance of Proteobacteria (Fig. 1D).

To investigate whether the relative abundance of any specific taxa significantly differed between the two populations, we performed a differential abundance analysis across taxonomic levels from phylum to genus. A total of 14 taxa including three orders, five families, and six genera significantly differed in relative abundance between populations (Fig. 2B). In addition to the shared taxa with significantly different abundances, the Ural population had 9 unique bacterial families including Campylobacteraceae, Moraxellaceae, Acetobacteraceae, Bacteroidales, Micrococcaceae, Peptostreptococcaceae, Coriobacteriales incertae sedis, M2PB4-65 termite group (order Bacteroidales) and p-2534-18B5 gut group (order Bacteroidales). The Betpak-Dala population had 3 unique families; Methanosarcinaceae, Oxalobacteraceae, and Microbacteriaceae. At genus level, the Ural population had 15 unique taxa; Campylobacter, Acinetobacter, Acetobacter, M2PB4-65 termite group incertae sedis, Alloprevotella, p-2534-18B5 gut group incertae sedis, Bacteroidales, Arthrobacter, Rummeliibacillus, Lactococcus, Eubacterium, Atopobium, Slackia, Romboutsia, and FD2005 (family Lachnospiraceae). Betpak-Dala population had 9 unique taxa; Methanimicrococcus, Rikenella, GWE2-31-10 (family Spirochaetaceae), Rathayibacter, Pseudoramibacter, Bacteroides pectinophilus group (family Lachnospiraceae), Oribacterium, 28-4 (family Lachnospiraceae).

The two Saiga populations had very similar ASV richness (Wilcoxon rank sum test, *p* = 0.978; estimated ASV richness range in Ural Saiga 475–994, mean 759, median 755; range in Betpak-Dala Saiga 498–1087, mean 763, median 761). Similarly, while the Ural Saiga had a slightly higher Shannon diversity index, this difference was not statistically significant (Wilcoxon rank sum test, *p* = 0.262; range in Ural Saiga 113–397, mean 245, median 257; range in Betpak-Dala Saiga 116–325, mean 227, median 232; Fig. 2C).

### Comparison between Saiga antelope and five other antelope species

The Saiga gut microbiota was compared with that of five other antelope species for which publicly available 16S rRNA microbiota data could be retrieved: the Tibetan antelope (*Patholops hodgsonii*)^23^, Przewalski’s gazelle (*Procapra przewalskii*)^24^, Impala (*Aepyceros melampus*)^18^, Springbok (*Antidorcas masupialis*)^18^, and Sable antelope (*Hippotragus niger*)^18^. The samples originated from China, South Africa, and Namibia (Suppl. Table 1). The investigated antelope species typically live in similar habitats, namely deserts, grasslands, and shrublands^3^. The target region of 16S rRNA gene varied across the datasets, thus all 16S amplicon sequences were trimmed to the V4 region for this comparative analysis to make them comparable (see “Methods”).

**Figure 3:**
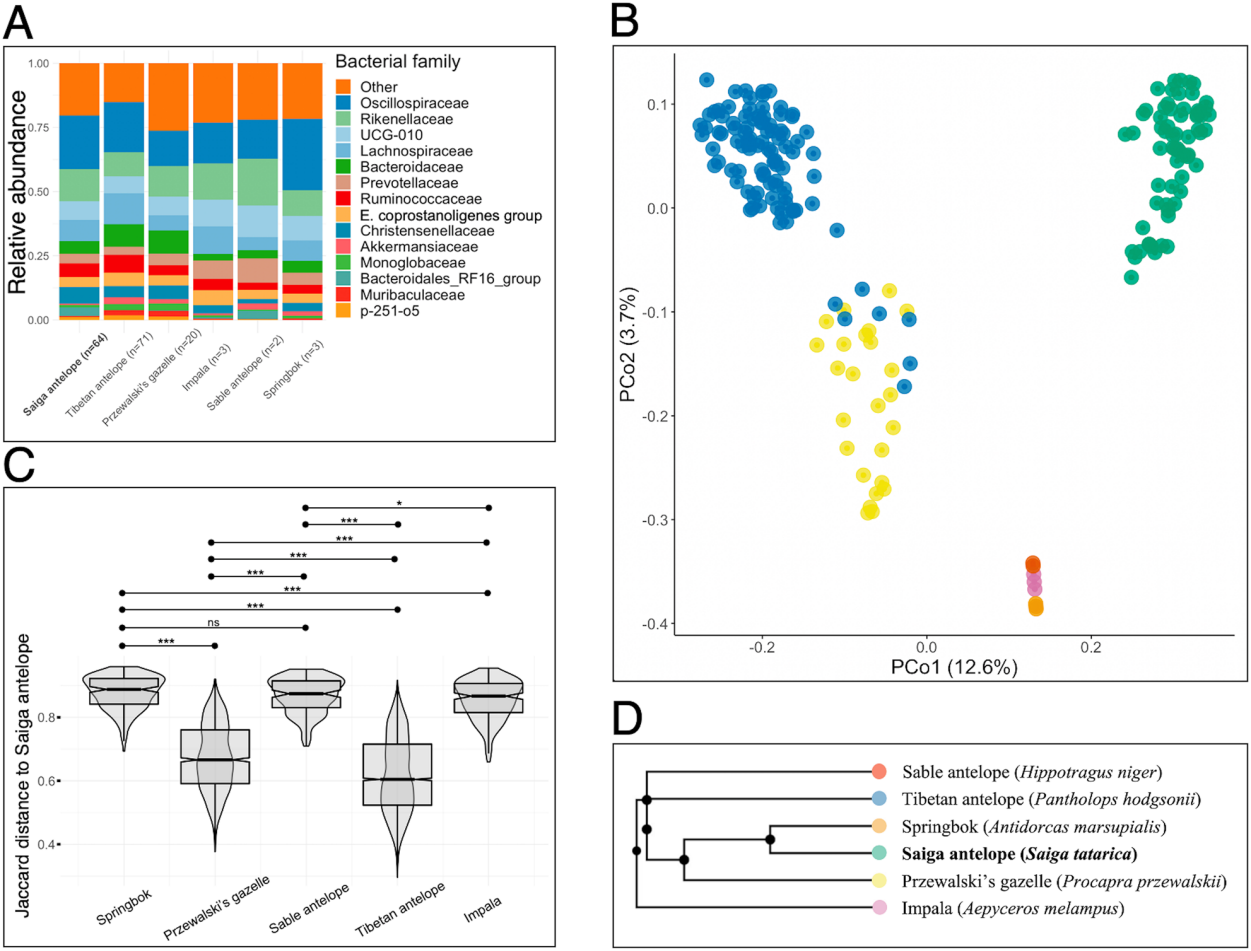
(**A**) The gut microbiota composition of six antelope species at family level. Rare taxa (mean relative abundance < 0.03% and prevalence < 0.10% across samples) and taxa for which bacterial family could not be assigned are under ‘Other’. (**B**) Principal coordinate analysis of gut microbiota (dis)similarity of six antelope species based on Jaccard distance. Taxa have been agglomerated to the family level before ordination. Circles are individual samples. Colour indicates host species (green, Saiga antelope; yellow, Przewalski’s gazelle; blue, Tibetan antelope; orange, Springbok; pink, Impala; red, Sable antelope). (**C**) Pairwise dissimilarity of bacterial genera (Jaccard distance) between Saiga antelope and (left to right) Springbok, Przewalski’s gazelle, Sable antelope, Tibetan antelope, or Impala (antelopes ordered by phylogenetic relatedness to Saiga antelope starting with closest relative). Differences were tested with permutational Wilcoxon rank sum tests (***, *p* < 0.001; *, *p* = 0.042; ns, *p* > 0.05). (**D**) A cladogram of six antelopes. The nodes indicate a common ancestor and the lines are relative to evolutionary timescale. The cladogram of the six antelopes was retrieved from the TimeTree database.

The gut microbiota composition of the Saiga was, at a high taxonomic level, broadly similar to that of the five other antelope species. The predominant phyla in all species were Firmicutes (54.1–70.9% of total abundance) and Bacteroidota (23.8–39.9%). At family level, Oscillospiraceae and Rikenellaceae formed the two predominant bacterial families (25.6–34.2%) in all antelope species except the Tibetan antelope, which had Oscillospiraceae and Lachnospiraceae as the most abundant families, followed by Rikenellaceae (Fig. 3A). At the ASV level, Saiga shared 13.6% and 14.2% of their ASVs with Tibetan antelope and Przewalski’s gazelle, respectively (Suppl. Fig. 4A), whereas no common ASVs were detected between Saiga (or the other two Asian antelopes, Przewalski’s gazelle and Tibetan antelope) and the African antelopes (Impala, Springbok, and Sable antelope).

Host species identity, which could not be distinguished from dataset identity, had a significant effect on gut microbiota composition explaining 24.1% of variation in bacterial community structure across the dataset (PERMANOVA based on Aitchison distance, *p* < 0.001; host species identity explained 19.5% of Jaccard dissimilarity, *p* < 0.001), although beta dispersion varied significantly among the datasets and could have affected these results (*F* = 458.74, *p* = 0.001). In ordination analyses, Saiga samples clustered separately from samples from all other antelopes on Jaccard, unweighted UniFrac and Aitchison distances but together with other samples on weighted UniFrac distance (Fig. 3B, Suppl. Fig. 4B–D).

The Saiga shared the majority of its gut microbial taxa with at least one antelope at all investigated taxonomic levels (phylum → family). It shared all of its 14 phyla with at least one other antelope species and 12 phyla (85.7%) with at least three other antelope species (Suppl. Fig. 5A). The other antelopes collectively harboured an additional eight phyla not found in the Saiga (Patescibacteria, Campilobacterota, Myxococcota, Nitrospirota, Acidobacteriota, Chloroflexi, Fusobacteriota, and Synergistota). At class and order levels, the Saiga shared 95.5% and 81.8% of its taxa, respectively, with at least three other antelope species. At family level, the Saiga shared 75.7% of taxa with at least three other antelopes (Suppl. Fig. 5B). These 56 bacterial families formed 94.2% of the total relative abundance of the Saiga gut microbiota. The Saiga harboured two unique bacterial families not detected in any of the other antelope species; Methanosarcinaceae (only detected in Betpak-Dala Saiga) and p-2534-18B5 gut group (order Bacteroidales; only detected in Ural Saiga). These families detected only in Saiga were rare, representing less than 1% of total relative abundance in both Saiga populations.

In terms of shared genera (Jaccard dissimilarity measured at genus-level, due to lack of shared ASVs between Asian and African antelopes), the Saiga microbiota was more similar in composition to that of other Asian antelope species (Tibetan antelope, Przewalski’s gazelle) than African species (Sable antelope, Springbok, Impala) (Fig. 3C, D). However, when considering the phylogenetic distance between genera (unweighted UniFrac distance on genera), the Saiga microbiota was more similar to that of African, rather than Asian, antelope species (Suppl. Fig. 6).

Compared to the other antelope species examined, Saiga had the highest alpha diversity (ASV richness and Shannon diversity; Suppl. Fig. 7). The difference in Shannon diversity was significant between Saiga and all other antelopes (Wilcoxon rank sum tests, *p* < 0.02 for all). However, the only significant difference in ASV richness was between Saiga and Tibetan antelope (Wilcoxon rank sum test, *p* < 0.001; *p* > 0.05 for all other comparisons).

## Discussion

The critically endangered Saiga antelope is a species of particular interest because of its vulnerability to mass mortality events. Together with anthropogenic factors, such as poaching and agricultural use, and linear infrastructure expansion, mass mortality events in Saiga can lead to the extinction of regional populations with observed losses of 75% and 88% of the affected population in 1988 and 2015, respectively^25^. A better understanding of the baseline biology of the Saiga could provide insights into their susceptibility to mass mortality events and tools for more successful husbandry in captivity^21,22^. As a step towards this, we present a gut microbiota profile of the Saiga, the first to our knowledge, from samples taken in a year without mass mortality and compare it to the gut microbiota of five other antelope species.

Similar to other mammals^26^, the gut microbiota of the Saiga was dominated by Firmicutes and Bacteroidota, which together formed 95% of total relative abundance. Two subpopulations of the Kazakh Saiga were sampled in the Betpak-Dala and Ural regions. These sampling locations were over 1000 km apart and the estimated geographic ranges of these migrating populations are at least 500 km apart^27^. Despite the geographic separation, the two populations shared approximately 85–94% of taxa at all inspected taxonomic levels from phylum to genus, and the shared genera formed over 80% of total relative abundance in both populations.

The Betpak-Dala and Ural Saiga populations displayed similar gut microbial alpha diversity, but differed in microbiota composition. Others investigating gut microbiota diversity across two wild populations of the Przewalski’s gazelle, a close relative of the Saiga, did not detect significant population differences in either alpha or beta diversity^24^. These populations are, however, geographically closer to each other (sampling locations < 200 km apart) than the Betpak-Dala and Ural Saiga populations (sampling locations > 1000 km apart), which could contribute to the greater cross-population similarity in the gut microbiota of Przewalski’s gazelle compared to Saiga. Geographical proximity could affect gut microbiota similarity through, for instance, similar vegetation type and thus diet as well as population mixing and gut microbe transmission^9,28^.

Due to the opportunistic nature of sampling and the tendency of the Saiga to avoid people, we were unable to collect more information concerning the individual animals. Hence, it remains unknown how much of the variation within and across the populations was driven by factors such as age and sex. Similarly, while various steps were taken to minimise the potential effect of exposure to oxygen and environmental microbes (see “Methods”), it is possible that the microbial composition of faecal matter was altered by these factors. Considering the two populations inhabit different areas at least 500 km apart, it is likely some of the gut microbial variation was driven by differences in habitat and diet, as has been found in other wild mammalian species^29^. The faecal samples used in the study were collected during or shortly after the Saiga calving period in May. Hence, the gut microbiota profiles reflect those in the calving period. While this might vary from the Saiga gut microbiota outside calving period, for example due to differences in hormone levels^30^, this is a relevant time point for sampling the Saiga since the previous mass mortality events have occurred during calving^1^, when the birthing females are likely to be stressed and potentially immunocompromised.

As a commensal, *Pasteurella multocida* (the putative causative agent of the Saiga mass mortalities) is most often found in the oral, nasopharyngeal, and respiratory microbiota^31^ and previous work has demonstrated the presence of the bacterium in the respiratory tract of healthy Saiga^32^. Analysis of tissue samples from the 2015 Saiga mass mortality event provided indication of a possible *P. multocida* invasion from the gut (the bacterium was detected in intestinal mucosa, alongside other body sites^1^), thus we searched for the presence of this bacterium in our dataset. We did not detect *P. multocida* in the Saiga gut microbiota and the closest taxonomic rank was the class Gammaproteobacteria.

The absence of *P. multocida* could be an artefact of the method since we characterised the gut microbiota by targeting a region of the 16S rRNA gene, a commonly used method that provides limited fine-scale taxonomic resolution, particularly at species level^33^. The method should provide a representative profiling at lower levels, such as at class and family levels^34^. Considering the class Gammaproteobacteria was the closest taxonomic rank detected to *P. multocida,* our 16S data does suggest the bacterium was not present in the Saiga gut microbiota in this data collected in a year without mass mortalities, or alternatively its abundance was below the detection threshold of the methods used.

To put the Saiga gut microbiota into a wider phylogenetic perspective, we compared it to that of five other antelope species (Tibetan antelope, Przewalski’s antelope, Sable antelope, Springbok, and Impala) for which 16S rRNA V4 gut microbiota data from wild individuals was publicly available. Saiga shared the majority of its taxa with other antelope species across taxonomic levels from phylum to genus. Still, microbiota compositions varied significantly between Saiga and the other antelopes. This could have been affected by environmental factors, such as geographical location and diet, as well as experimental factors, such as DNA extraction kit and sequencing batch, which varied between the datasets and could not be controlled for due to small sample size^28,35,36^. The finding that the hosts shared a high number of taxa at the various taxonomic levels despite these methodological limitations suggests the Saiga does not present an outlier within antelopes from the gut microbiota perspective.

In terms of shared taxa, the Saiga gut microbiota was more similar in composition to antelopes from the same rather than different continent when similarity was measured with non-phylogenetically informed Jaccard distance. However, this conclusion depended on the distance metric considered; when a phylogenetic distance metric was used (unweighted UniFrac), the Saiga microbiota was most similar to that of an African antelope species and its closest relative (Springbok), rather than geographically more proximate Asian antelope species. These results suggest that phylosymbiosis, under which microbiota similarity is expected to correlate with phylogenetic relatedness and which is observed in several^37–40^ but perhaps not all animals^41^, may be detected in the studied antelope species only when phylogenetic distance of gut microbes is considered. When phylogenetic relatedness of microbes was not considered, geography rather than host phylogeny appeared to have a stronger influence on Saiga microbiota in relation to other antelopes.

Overall, our results indicate the gut microbiota of two geographically disparate Saiga antelope populations is taxonomically rather consistent, but varies in relative abundance of bacterial taxa. We did not detect *Pasteurella multocida*—the bacterium thought to cause Saiga mass mortalities—in the Saiga gut microbiota during this year without die-offs. Finally, we showed that the Saiga gut microbiota resembles that of other antelopes. With this, we provide a baseline description of the gut microbiota in this critically endangered species, on which future work examining the potential role of the microbiota in mass mortality events can build.

## Methods

### Sample collection

Faecal samples were used to provide a non-invasive characterisation of the gut microbiota. Faecal samples of Saiga antelope were collected opportunistically from two populations in north-west (within 25 kms from 49°59′ N, 47°40′ E; ‘Ural population’) and central (within 25 kms from 49°30′ N, 61°51′ E; ‘Betpak-Dala population’) Kazakhstan in May 2019, which is during and immediately after the peak calving period.

Faecal samples were collected in a non-intrusive manner. Saiga individuals were located and observed (either directly or through binoculars) until defecation and once they moved on from that location, a fresh faecal sample was collected. The Saiga is the only wild ungulate in the region, with distinct pellet-shaped faeces, and tends to avoid livestock^42^, minimising the risk of faeces mis-identification. During sampling, faecal matter was picked up using clean disposable gloves and dissected with sterile forceps in order to get two separate 100 mg aliquots from inside faecal pellets, to avoid environmental contamination. The time between defecation and collection of samples was within an hour in most cases. While it was not possible to ensure that every faecal sample was from a different individual as Saiga were not marked, our collection methodology made it unlikely to sample the same individual twice: faecal sampling was conducted at various locations over several days within the two sites (Ural, Betpak-Dala) where tens of thousands of Saigas had gathered to calve.

Faecal samples were immediately preserved in DNA/RNA Shield, a preservation solution that protects DNA against degradation and allows sample storage at ambient temperature (Zymo Research, Irvine, California, USA). After a maximum of 3 weeks at ambient temperature in the field, samples were kept at − 20 °C in Kazakhstan until shipping at ambient temperature to the UK in March 2020 (under an import permit from the UK Plant and Animal Health Agency). Upon receipt samples were kept at − 80 °C until DNA extraction.

### DNA extraction, PCR amplification, and amplicon sequencing

Genomic DNA was extracted using the ZymoBIOMICS DNA Miniprep Kit, following the manufacturer’s protocol (Zymo Research, Irvine, California, USA). A total of 80 samples (32 samples from the Ural population and 48 samples from the Betpak-Dala population) were randomised into four extraction batches. The 80 samples included 10 duplicate aliquots which were used to check the robustness of our sample and data processing pipeline. DNA was eluted in 50 μL DNAse-free H_2_O, and one negative extraction control (DNAse-free H_2_O) was included in each extraction batch. Library preparation and sequencing was conducted at the Integrated Microbiome Resource, Dalhousie University, as described in Comeau et al.^43^. The V4–V5 region of the 16S rRNA gene was amplified by PCR using the 515F–926R primers^44,45^. All samples were amplified and sequenced in one batch using the Illumina MiSeq platform (Reagent kit v3, 2 × 300 bp chemistry). The sequencing run included a negative control for the PCR reaction and a negative control for the sequencing. All four extraction controls were sequenced either on the sequencing run in question or on subsequent sequencing runs.

### Online data acquisition

A Web of Knowledge search was conducted in November 2021 to identify datasets which would allow comparison of the Saiga gut microbiota to that of other wild antelope species. Search keywords included *gut microbiome, gut microbiota, antelope*, and *ungulate.* Studies of captive animals were excluded. Only publicly available datasets for which V3–V4, V4, or V4–V5 16S rRNA primers and the Illumina sequencing platform had been used were considered. The retrieved and included datasets include the following five antelope species: Tibetan antelope (*Patholops hodgsonii*), Przewalski’s gazelle (*Procapra przewalskii*), Impala (*Aepyceros melampus*), Springbok (*Antidorcas masupialis*), and Sable antelope (*Hippotragus niger*) (Suppl. Table 1).

### 16S data processing

To make all datasets fully comparable, publicly available antelope datasets were downloaded and the raw sequencing reads were processed together with the Saiga sequencing reads using a standardized pipeline, as follows. Downloaded files were converted to match a format that was compatible with Quantitative Insights into Microbial Ecology (QIIME2, 2020.11 distribution) (zipped fastq files with a unique name). First, FastQC v0.11.9^46^ and MultiQC v1.12^47^ were used to visualize read quality, before cutadapt v3.4^48^ was used to remove adapters and/or primers where still present. Due to differences in targeted 16S rRNA region(s) (Suppl. Table 1), for comparative analyses across antelope species, sequencing reads were trimmed to include the V4 region (515F-806R) only to make amplicon sequence variants comparable using cutadapt^48^. In analyses of Saiga microbiota samples alone, such trimming was not performed, in order to provide as much resolution as possible.

Trimmed or original reads containing V4 region were then processed as follows. Low-quality reads were filtered using qiime2 quality-filter q-score (default settings, QIIME2 2020.11) before using the Deblur workflow^49^ to denoise sequences into ASVs, as suggested by QIIME2^50^. Within Deblur, trimming length was determined by manually viewing the quality plot for each study. ASV taxonomy was assigned using a classifier trained on the full-length 16S rRNA gene SILVA v138.1 database^51^, and ASVs taxonomically assigned as ‘mitochondria’ or ‘chloroplast’ as well as those not identifiable at the kingdom level were removed. Phylogenetic trees of the remaining ASVs were built using the SEPP qiime2 plugin (https://github.com/bioshared/q2-fragment-insertion) with a reference phylogeny (sepp-refs-gg-13-8).

Negative controls for DNA extraction (*n* = 4) and library preparation (*n* = 1) of Saiga antelope samples collectively contained 13 ASVs with a maximum read count for any given ASV per control of 9. The R package decontam^52^ was used to test for potential contaminants in the Saiga dataset, for which negative controls were available. The decontam test was conducted using the ‘prevalence’ (presence/absence) method, which compares each sequence in biological samples to the prevalence in negative extraction and PCR controls. A sequence was considered a contaminant if it reached a probability of 0.1 in the Fisher’s exact test used in decontam. No potential contaminants were identified in the Saiga dataset with this method.

Sample completeness and rarefaction curves were generated with R package iNEXT^53,54^ for all included datasets combined and the read depth threshold (below which samples were excluded from further analysis) was set at 4000, based on where these curves plateaued. Data were not rarefied. Singletons and doubletons (ASVs with a total of either one or two sequences across the dataset) were removed prior to beta (not alpha) diversity analyses to guard against the possible influence of remaining contaminants and sequencing errors. Microbiome profiles from duplicate Saiga samples were inspected using a principal coordinate analysis (PCoA) based on Aitchison distance in package vegan^55^, and the effect of sample ID for microbiota composition was tested using permutational multivariate analysis of variance (PERMANOVA) on Aitchison distance. Prior to ordination on Aitchison distance, a centered log-ratio (clr) transformation was performed using the package microbiome^56^, with relative abundance values of zero replaced with a pseudocount (min(relative abundance/2)). Duplicates were removed from the dataset before further analysis.

### Analyses

Data was analysed and visualised in R (v4.1.2) using packages phyloseq^57^, vegan^55^, microbiome^56^, ALDEx2^58^, UpSetR^59^, and ggplot2^60^. For the inspection and comparison of taxonomic compositions, ASV counts were transformed into relative abundance per sample. We searched for the presence of *Pasteurella multocida* in the Saiga gut microbiota both by investigating taxonomic assignments from the SILVA database as well as by using the NCBI Nucleotide Basic Local Alignment Search Tool (Nucleotide BLAST). For the latter, we BLAST searched all ASVs either assigned to Gammaproteobacteria (the bacterial class containing *P. multocida*) or not assigned a class by SILVA, against the *P. multocida* type sequence (taxid: 747).

Differential abundance testing of bacterial taxa across the two Saiga populations was conducted using package ALDEx2^58^. Monte-Carlo sampling (mc.samples = 128) from a Dirichlet distribution was used to generate a distribution of clr transformed values for all taxa. Welch’s and Wilcoxon rank sum tests were then performed on the clr transformed values. Taxa with a Benjamini–Hochberg corrected p-value (q-value) of less than 0.05 and an effect size greater than 1 were considered to have a significantly different abundance between the two populations. All taxa found in the Saiga data were included in the differential abundance testing, regardless of whether they are unique to one population or not. Asymptotic alpha diversity (ASV richness and Shannon diversity) was estimated using package iNEXT^53,54^. Differences in alpha diversity and Jaccard dissimilarity among samples from each population were tested using Wilcoxon rank sum tests.

Beta diversity metrics (Jaccard, Aitchison, unweighted UniFrac, weighted UniFrac) were calculated with the package vegan, and PERMANOVA was performed using Aitchison and Jaccard distances. Ordination was conducted with the package vegan using PCoA. A clr transformation was conducted before ordination for Aitchison distance (zero relative abundances replaced with pseudocounts as described in *Data processing*). Ordination plots were produced using the phyloseq package^57^. A cladogram showing the phylogenetic relationships among the six antelope species was retrieved from the TimeTree database.

### Ethics declaration

Faecal samples were collected non-invasively with minimal disturbance to animals.

### Supplementary Information

Supplementary Information.

## Data Availability

The 16S rRNA amplicon sequencing data of the Saiga antelope have been deposited in GenBank under SRA accession: PRJNA982717.
