# Supplementary material for: Gut microbiota of the critically endangered Saiga antelope across two wild populations in a year without mass mortality
Source: Sci Rep. 2023 Oct 11;13:17236. doi: 10.1038/s41598-023-44393-z (PMC10567781; doi:10.1038/s41598-023-44393-z)

**Supplementary material**


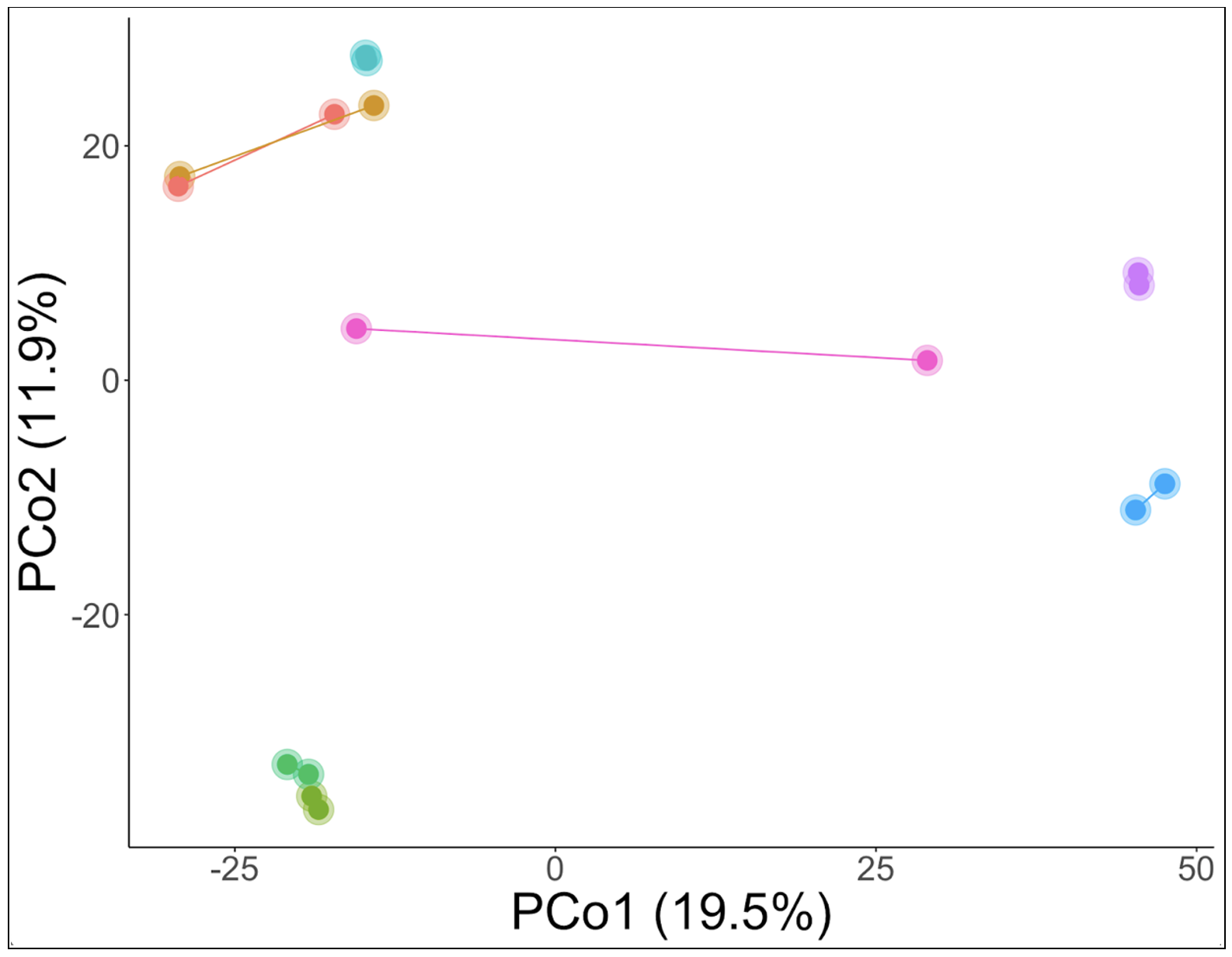


**Supplementary Figure 1.** Principal coordinate analysis (PCoA) on Aitchison distance for eight Saiga samples for which duplicates were included. Colour indicates sample ID (duplicates for each, connected with lines).


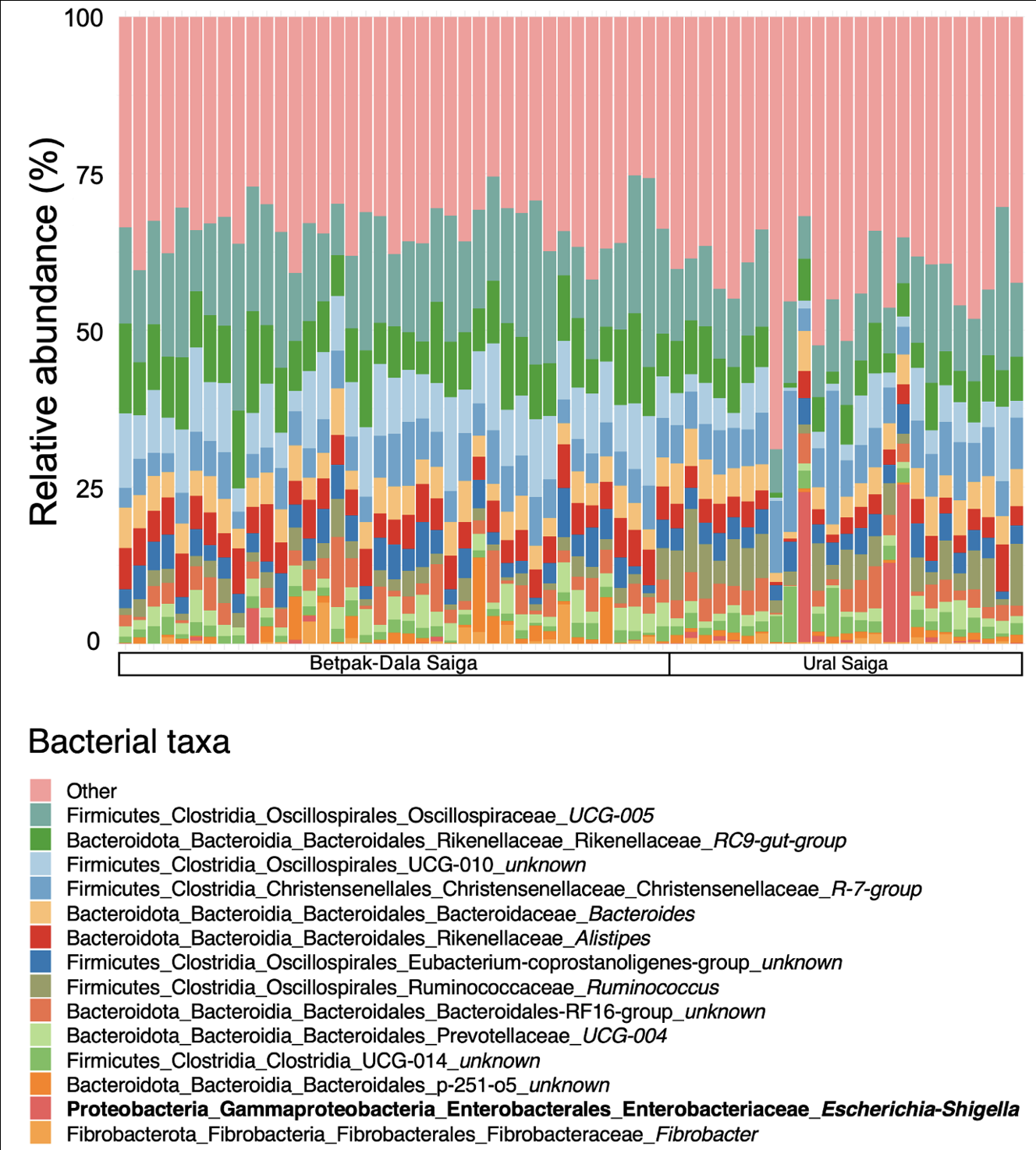


**Supplementary Figure 2.** Gut microbiota composition of the Saiga at family level. Stacked bars represent individual samples with horizontal bars indicating Saiga population. Rare taxa (mean relative abundance <0.03% and prevalence <0.10% across samples) and taxa for which bacterial family could not be assigned are under ‘Other’.


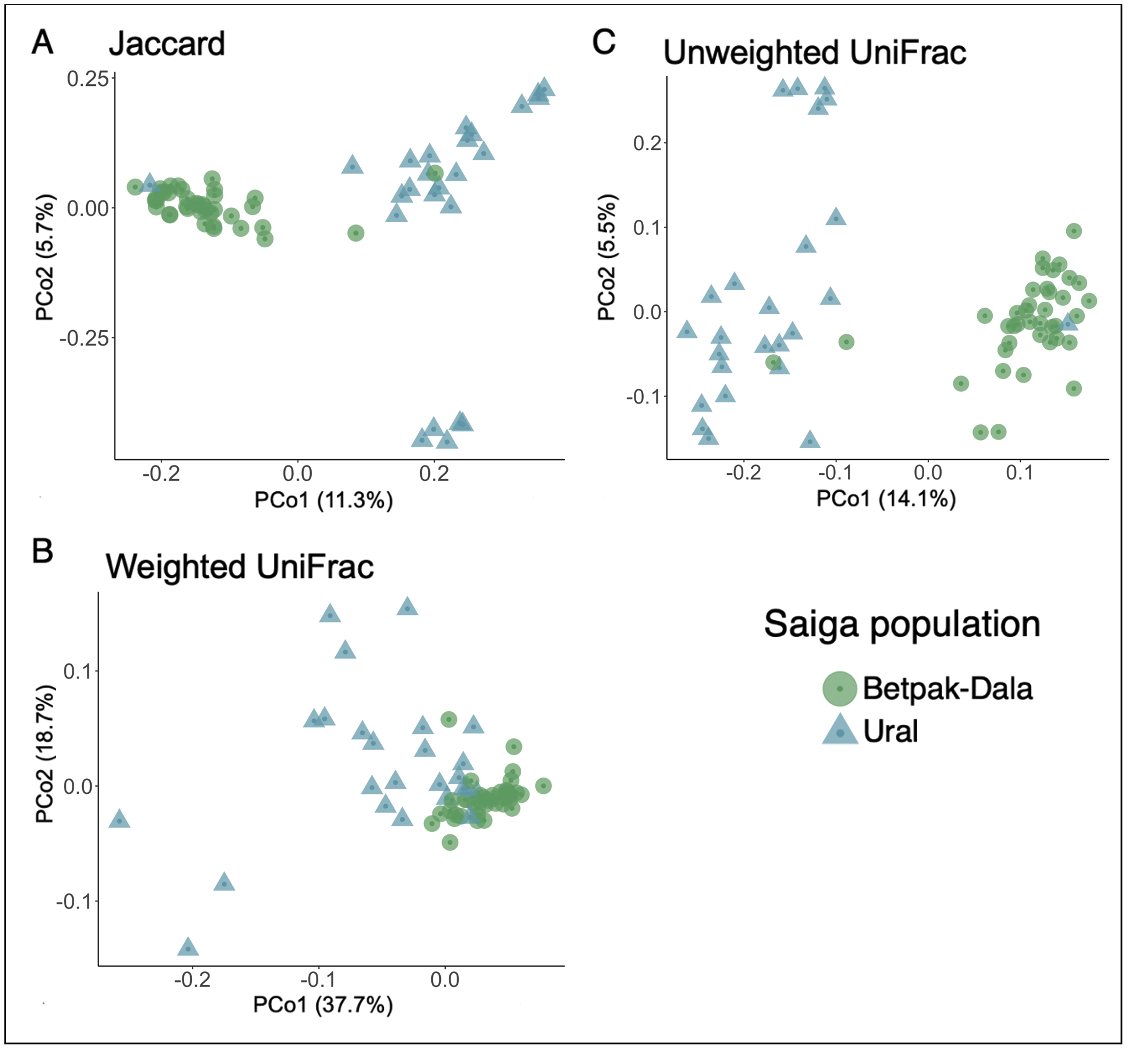
**Supplementary Figure 3.** Principal coordinate analysis of Betpak-Dala (*green*) and Ural (*blue*) Saiga gut microbiota on (**A**) Jaccard, (**B**) weighted UniFrac, and (**C**) unweighted UniFrac distances. Circles and triangles are individual samples.


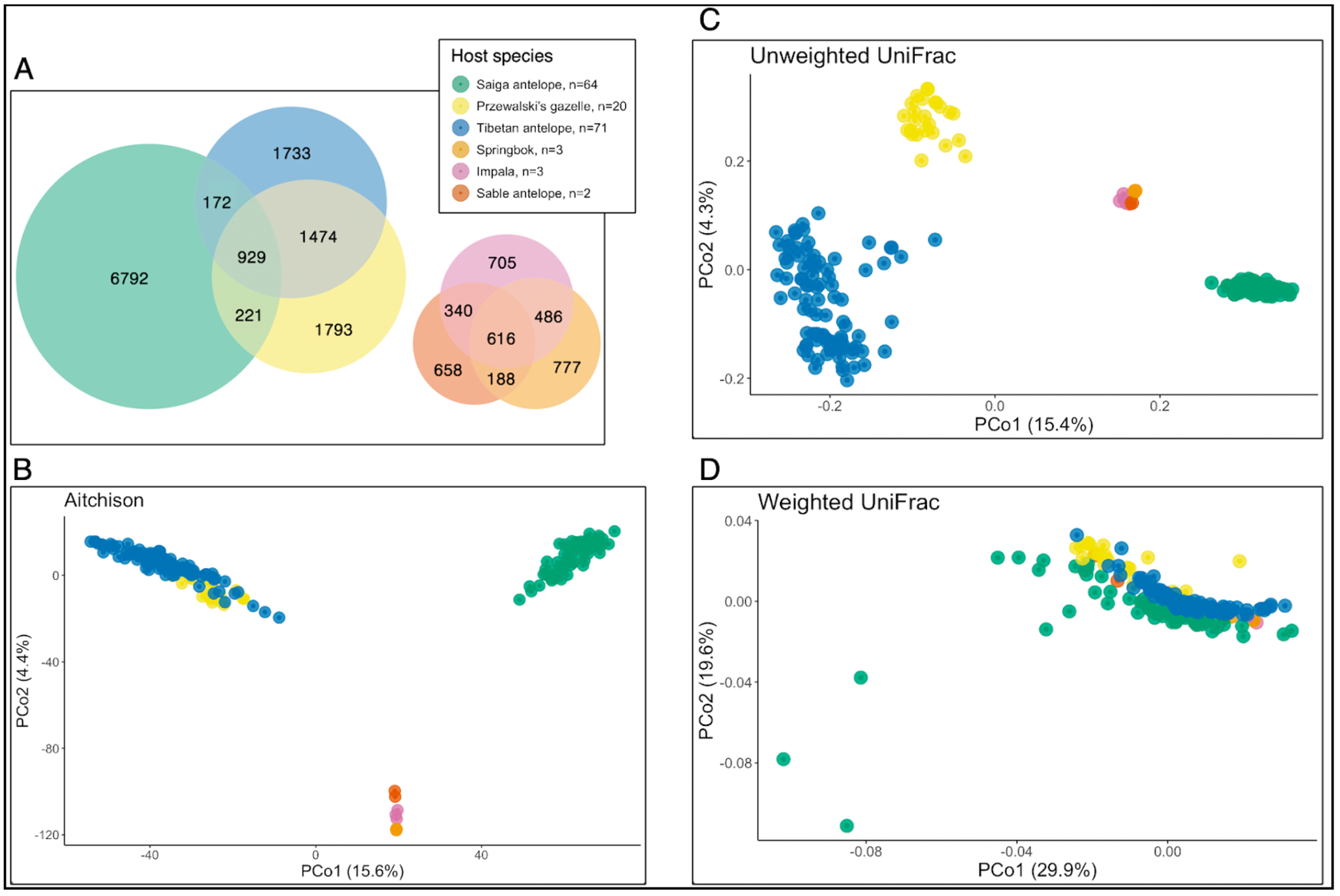


**Supplementary Figure 4.** (**A**) Euler diagram on shared and unique amplicon sequence variants in gut microbiota of six antelope species. (**B-D**) Principal coordinate analysis of gut microbiota (dis)similarity of six antelopes at amplicon sequence variants (ASV) level based on (**B**) Aitchison, (**C**) unweighted UniFrac, and (**D**) weighted UniFrac distances. Circles are individual samples. Colour indicates host species (*green* = Saiga antelope, *yellow* = Przewalski’s gazelle, *blue* = Tibetan antelope, *orange*= Springbok, *pink* = Impala, *red* = Sable antelope).


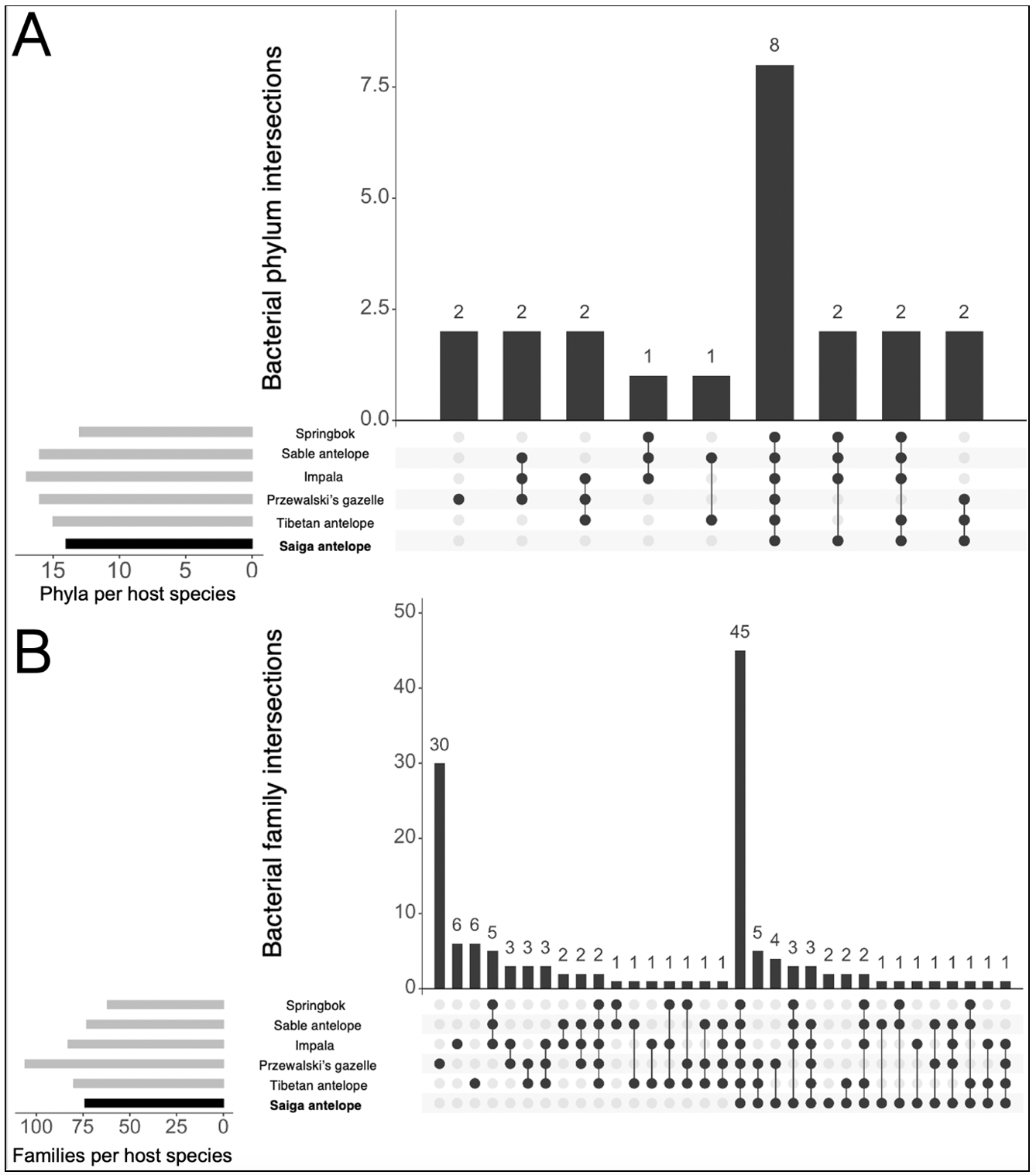


**Supplementary Figure 5.** UpSet plots showing quantitative intersections of bacterial (**A**) phyla and (**B**) families between six antelopes. The numbers above the vertical bars indicate the number of common taxa between host species.


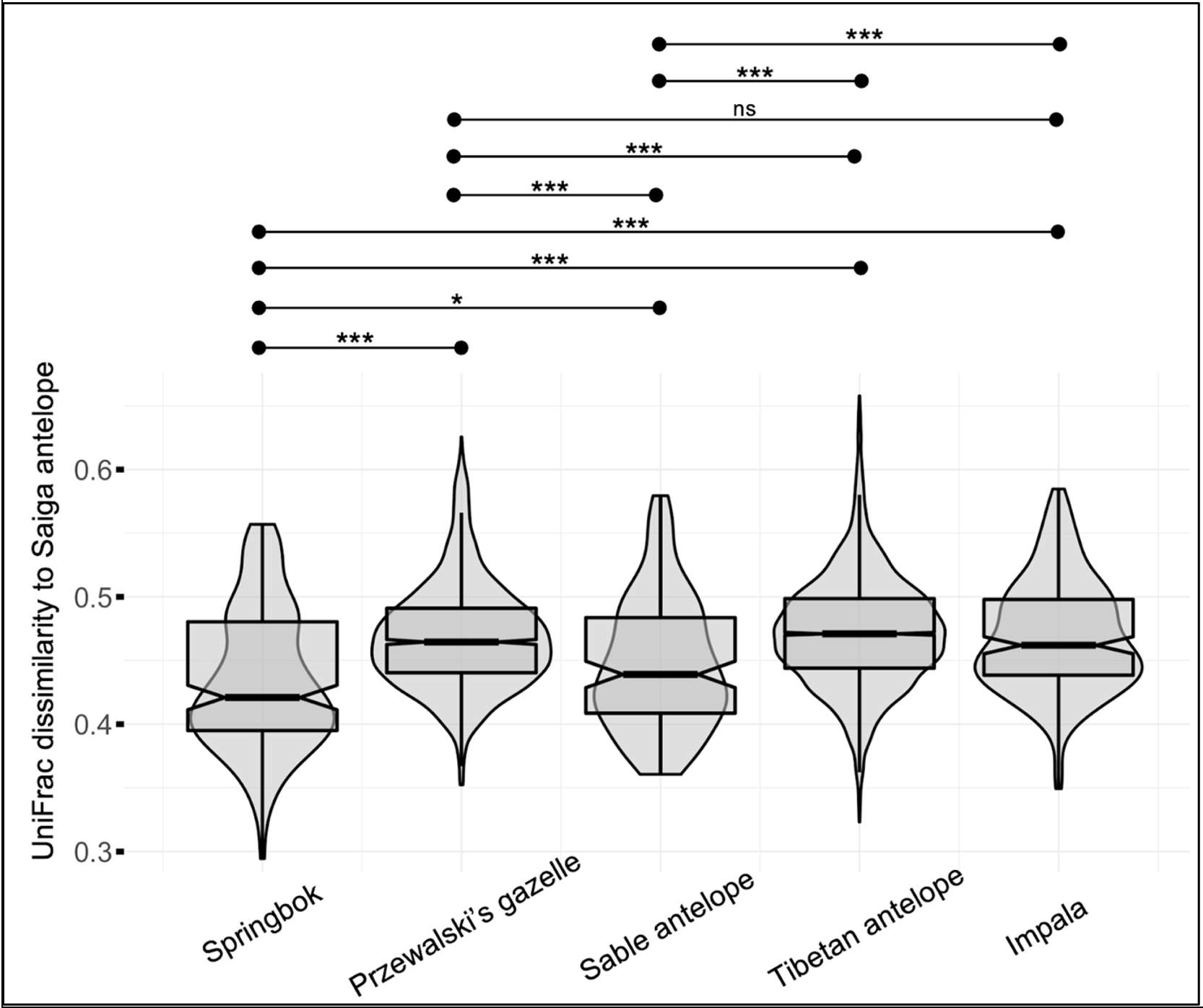


**Supplementary Figure 6.** Pairwise dissimilarity of bacterial genera (unweighted UniFrac distance) between Saiga antelope and (from left to right:) Springbok, Przewalski’s gazelle, Sable antelope, Tibetan antelope, or Impala (antelopes ordered by phylogenetic relatedness to Saiga antelope starting with closest relative). Antelope species are ordered by phylogenetic relatedness to Saiga with alphabetical ordering where relatedness to Saiga cannot be distinguished (Sable antelope, Tibetan antelope). Differences were tested with permutational Wilcoxon rank sum tests (***, *p*<0.001; *, *p*=0.034; ns, *p*=0.998).


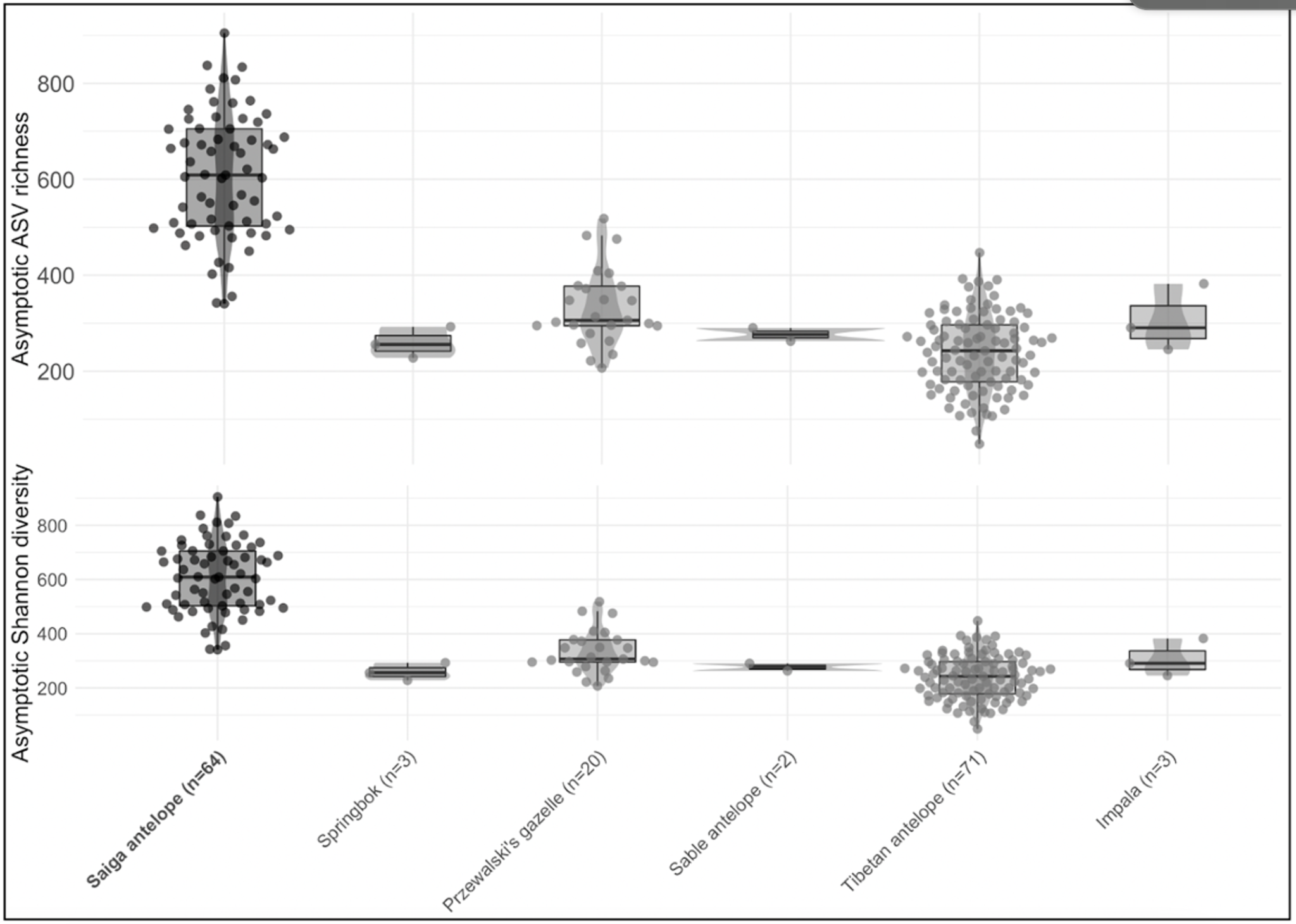


**Supplementary Figure 7.** Sample-level asymptotic estimates of ASV richness (*top*) and Shannon diversity (*bottom*) for six antelope species. Circles are individual samples. Antelope species are ordered by phylogenetic relatedness to Saiga with alphabetical ordering where relatedness to Saiga cannot be distinguished (Sable antelope, Tibetan antelope).

**Supplementary Table 1.** Datasets included in cross-host species analyses.


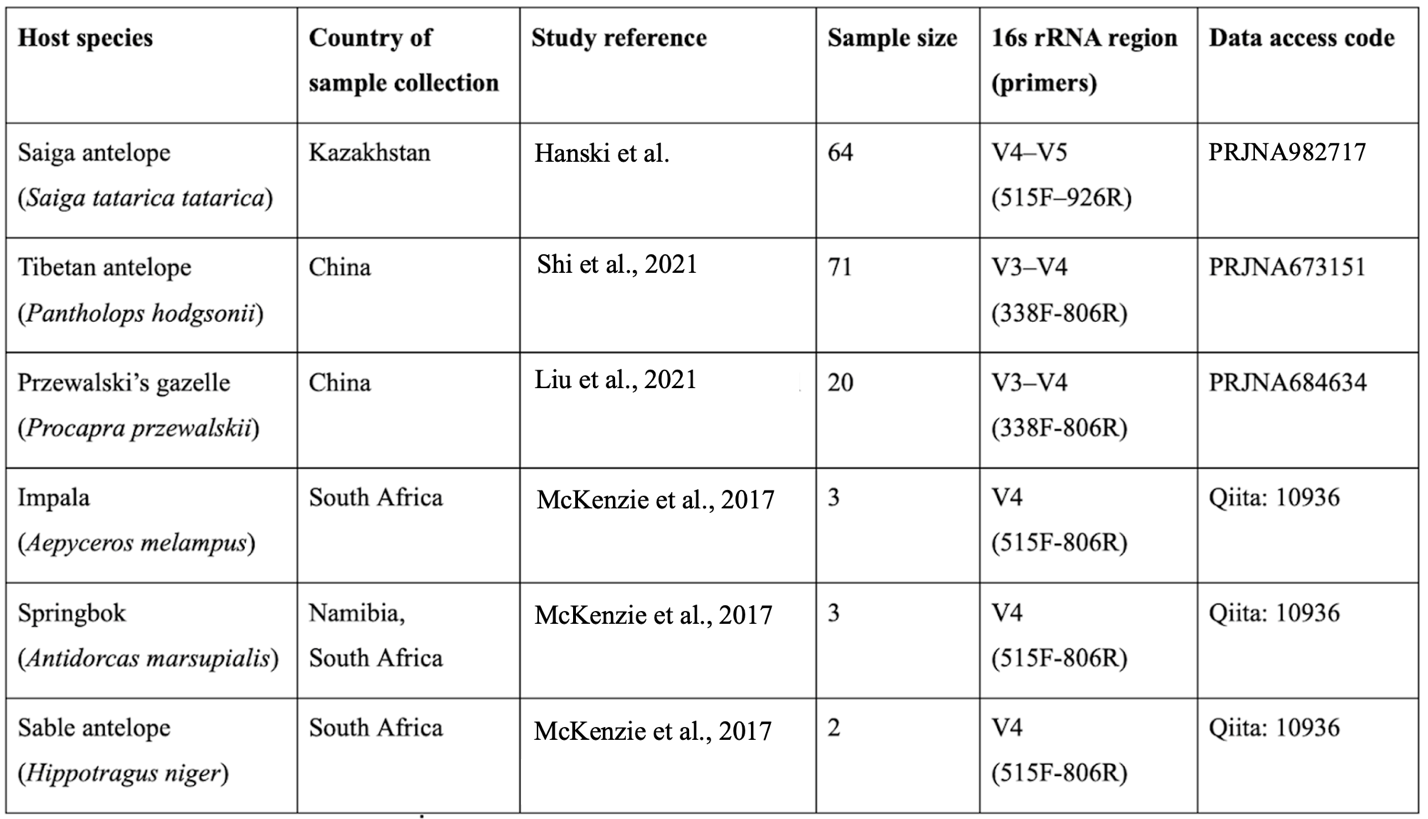

Supplement: Supplementary file 1 — Supplementary Information. [file 41598_2023_44393_MOESM1_ESM.docx]
